# Nrf2 as a target for prevention of age‐related and diabetic cataracts by against oxidative stress

**DOI:** 10.1111/acel.12645

**Published:** 2017-07-19

**Authors:** Xiu‐Fen Liu, Ji‐Long Hao, Tian Xie, Tayyab Hamid Malik, Cheng‐Bo Lu, Cong Liu, Chang Shu, Cheng‐Wei Lu, Dan‐Dan Zhou

**Affiliations:** ^1^ Department of Ophthalmology The First Hospital of Jilin University Jilin China; ^2^ Department of Neurosurgery The People's Hospital of Jilin Province Jilin China; ^3^ Department of Gastroenterology The First Hospital of Jilin University Jilin China; ^4^ Department of Cardiology The First Hospital of Jiamusi University Heilongjiang China; ^5^ Department of Obstetrics and Gynecology The First Hospital of Jilin University Jilin China; ^6^ Department of Radiology The First Hospital of Jilin University Jilin China

**Keywords:** antioxidant response element, cataracts, Keap1, lenses, Nrf2, oxidative stress

## Abstract

Cataract is one of the most important causes of blindness worldwide, with age‐related cataract being the most common one. Agents preventing cataract formation are urgently required. Substantial evidences point out aggravated oxidative stress as a vital factor for cataract formation. Nuclear factor (erythroid‐derived 2)‐like 2 (Nrf2)/Kelch‐like erythroid‐cell‐derived protein with CNC homology (ECH)‐associated protein 1 (Keap1) system is considered as one of the main cellular defense mechanisms against oxidative stresses. This review discusses the role of Nrf2 pathway in the prevention of cataracts and highlights that Nrf2 suppressors may augment oxidative stress of the lens, and Nrf2 inducers may decrease the oxidative stress and prevent the cataract formation. Thus, Nrf2 may serve as a promising therapeutic target for cataract treatment.

**Abbreviations**
AKT: serine–threonine kinase
ALCAR: acetyl‐l‐carnitine
ARCs: age‐related cataracts
ARE: antioxidant response element
ATF4: activating transcription factor 4
ATF6: activating transcription factor 6
BiP: immunoglobulin heavy chain binding protein
CHOP: CCAAT/enhancer‐binding protein‐homologous protein
eIF2α: eukaryotic initiation factor 2α
ER: endoplasmic reticulum
ERK: extracellular signal‐regulated kinase
GR: glutathione reductase
GSH: glutathione
GST: glutathione‐S‐transferase
H_2_O_2_: hydrogen peroxide
Hcy: homocysteine
HLECs: human lens epithelial cells
HO‐1: heme oxygenase‐1
IRE1α: Inositol‐requiring kinase 1α
Keap1: Kelch‐like erythroid‐cell‐derived protein with CNC homology (ECH)‐associated protein 1
LECs: lens epithelial cells
MAPK: mitogen‐activated protein kinases
MGO: methylglyoxal
NBP: DL‐3‐n‐butylphthalide
Nrf2: transcription factors like nuclear factor (erythroid‐derived 2)‐like 2
O_2_: oxygen
PERK: protein kinase RNA (PKR)‐like endoplasmic reticulum kinase
PI3K: phosphatidylinositol 3‐kinase
RLM: Rosa laevigata Michx
ROS: reactive oxygen species
SFN: sulforaphane
STZ: streptozocin
TrxR: thioredoxin reductase
Trx: thioredoxin
UPR: unfolded protein response
VPA: Valproic acid

## Introduction

## Oxidative stress and cataracts

### Oxidative stress

Oxidative stress is the outcome of instability of pro‐oxidants and antioxidants (Babizhayev & Yegorov, 2016). Decrease in molecular oxygen (O_2_) metabolites is considered as ‘reactive oxygen species’ (ROS) on account of the higher reactivity in relation to molecular O_2_. ROS are mainly short‐lived and highly reactive molecules, which are induced intracellularly through different pathways, such as by‐products during normal aerobic metabolism or as messengers in different cellular signaling pathways. In addition, ROS can also be produced by cells from exogenous sources, either being generated as a result of the cells’ experience with several environmental stimuli or absorbed instantly from the extracellular milieu (Babizhayev & Yegorov, 2016). Oxidative stress is important to regulate normal physiological functions associated with cell cycle, migration, and cell death (Redza‐Dutordoir & Averill‐Bates, 2016; Schumann *et al*., 2016). Oxidative stress has been involved in the activation of different transcription factors like nuclear factor (erythroid‐ derived 2)‐like 2 (Nrf2, also known as NFE2L2)/Kelch‐like erythroid‐cell‐derived protein with CNC homology (ECH)‐associated protein 1 (Keap1), mitogen‐activated protein kinases (MAPK), which are involved in the activation of cell survival and death processes. On the contrary, when cell oxidant production overwhelms the intrinsic antioxidant detoxification capacity, damage to cellular macromolecules like lipids, proteins, nucleic acids, membranes occurs. Oxidative stress is believed to involve in the pathogenesis of various aging‐related diseases, and sight‐threatening eye diseases (Babizhayev & Yegorov, 2016). Clinical evidence supports a connection role of oxidative stress with diseases, but the exact underlying molecular mechanisms are still not entirely understood.

### Cataract vs. oxidative stress

Human lens is composed of lens capsule (outer layer), lens epithelium (middle layer), and lens fibers (inner layer). Human lens epithelium is composed of a monolayer of lens epithelial cells (LECs) with foremost metabolically activities (such as oxidation, cross‐linking). LECs migrate to the equator portion of the lens, transform into lens fibers, gradually compress centrally, and form the nuclear opacity with aging increase.

Cataracts, the opacification of eye lens, are the foremost cause of blindness worldwide. Aging is the foremost cause of the cataracts, followed by other causes such as environmental factors (trauma, radiation exposure etc.), genetic susceptibility, and ocular diseases (Liu *et al*., 2017b). Age‐related cataract can be divided into cortical (wedge‐shaped, starting from the cortex and extending to the center), posterior subcapsular (plaque‐like, locating in the axial posterior cortical layer), and nuclear cataracts according to the opacity location. Ultraviolet irradiation and oxidative damage are considered as predominant contributors for cataract formation. Aging eye seems to be at great risk to oxidative stress (Babizhayev & Yegorov, 2016), and LECs are inclined to ROS (Vinson, 2006; Bassnett *et al*., 2011). Human lens consists of α, β, and γ crystalline proteins, and oxidative stress may lead to aggregate of these proteins, developing clumps, and results in loss of transparency with increasing age, leading to cataract formation. Besides oxidation of crystalline proteins, DNA damage, membrane lipid peroxidation, and imbalance in calcium homeostasis all contribute to the oxidative stress‐induced cataract formation. Cataracts also are prevalent in diabetes where the level of superoxide in the mitochondria is increased owing to hyperglycemia (Vinson, 2006).

Although cataract surgery has been considered very successful, surgery‐associated complications unavoidably cause irreversible blindness. Thus, it is imperative to identify strategies and antioxidants that would prevent cataract formation based on the association between cataract and oxidative stress; natural compounds namely vitamin C, vitamin E, and curcumin have been considered as promising antioxidants in preventing the cataract formation.

## Nrf2‐Keap1 system and cataracts

### Classic Nrf2‐Keap1 system and oxidative stresses

Nrf2‐Keap1 system is known as one of the main cellular defense mechanisms against oxidative stresses. Nrf2 is a vital nuclear transcriptional inducer (Cullinan & Diehl, 2004), which binds to the antioxidant response element (ARE) in the DNA promoter and controls the transcriptions of many antioxidant genes, including glutathione‐S‐transferase (GST), glutathione reductase (GR), thioredoxin reductase (TrxR) (Rushmore *et al*., 1991; Yu *et al*., 2010). Regulated by protein kinase RNA (PKR)‐like endoplasmic reticulum kinase (PERK) (Cullinan & Diehl, 2004), MAPK (Yu *et al*., 2000), protein kinase C (Bloom & Jaiswal, 2003), phosphatidylinositol 3‐kinase (PI3K), etc. (Kang *et al*., 2002), Nrf2 serves as a molecular switch turning on/off of the Nrf2‐mediated antioxidant systems. Keap1 serves as an oxidative stress sensor and a primary Nrf2 inhibitor. The promoter of *Keap1* gene contains a key CpG island, with 68 CpG dinucleotides in total, and locates between −460 and +341. DNA demethylation in the *Keap1* promoter eventually disturbs the Nrf2/Keap1‐dependent antioxidant protection (Palsamy *et al*., 2012). Keap1 constantly targets Nrf2 for ubiquitination and subsequent 26S proteasomal degradation, maintaining Nrf2 at basal level. Under favorable conditions, Nrf2 location is restricted to the cytoplasm by binding with Keap1 in the form of Nrf2/Keap1 complex. Under stressed conditions, such as oxidative or endoplasmic reticulum (ER) stress, Nrf2 separates from Keap1, phosphorylates, and translocates into the nucleus, and induces the ARE‐controlled antioxidant gene transcription, which initiates detoxification of ROS through regulation of GSH (Nakagami, 2016).

### Nrf2‐Keap1 system and cataracts

Aging is a key risk factor for most disorders such as cataracts and the causes remain elusive. ROS, which may cause the oxidative damage to proteins, lipids (e.g., lipid peroxidation), and DNA (e.g., oxidation, methylation), has been assigned to be one of the main damage concepts to the aging. Senescent cells those have lost the ability to divide contribute to age‐related diseases, and their selective removal increases physiological function and extends longevity (Naylor *et al*., 2013). Caloric restriction may enhance lifespan and delay age‐related diseases (Bishop & Guarente, 2007). As one of the major regulators of system defense against the oxidative stress, Nrf2 is also a vital regulator of the beneficial effects of calorie restriction, and it correlates with the increased autophagy and decreased induction of senescence cell. Nrf2 and its target genes showed a decrease with age in rats (Shih & Yen, 2007). The levels of total as well as nuclear Nrf2 protein were drastically lower in the liver of older vs. younger rats (Suh *et al*., 2004), indicating that Nrf2 is a protective factor during aging. Tomatidine, abundant in tomatoes, extended lifespan and healthspan in *Caenorhabditis elegans* (an animal aging model sharing vital longevity related pathways with mammals) via the Nrf2 pathway (Fang *et al*., 2017). Nrf2 activator protandim, a botanical extracts (Velmurugan *et al*., 2009), increased longevity in mice (Strong *et al*., 2016), and orally protandim supplements robustly elevated superoxide dismutase (SOD) and catalase level in erythrocyte of healthy humans (Nelson *et al*., 2006). Fumarate, the citric acid cycle metabolite provided cardioprotection via Nrf2 activation in mice (Ashrafian *et al*., 2012). Nrf2 also mediates cancer protection induced by caloric restriction (Pearson *et al*., 2008). The long‐lived species (e.g., naked mole rat) may rapidly respond and neutralize the stressors (e.g., endogenous ROS and environmental stressors), retarding the age‐related diseases (e.g., cataracts, neurodegeneration, cardiovascular diseases, cancers). Keap1 and β‐transducin repeat‐containing protein are both negative Nrf2 regulators which target Nrf2 for degradation, and their different expression profiles may contribute the divergent longevity of species (Lewis *et al*., 2015). These results indicate that Nrf2 activation may confer stress and extend lifespan, motivating researches into delaying and preventing aging.

Rapamycin is an mTOR inhibitor, and rapamycin‐based therapies have been studies for combating aging (Arriola Apelo & Lamming, 2016). Rapamycin extended lifespan in mice with retarded age‐associated activity declining and postponed cancer caused death (Harrison *et al*., 2009; Miller *et al*., 2011). RNA interference against mTOR in *C. elegans* extended worm lifespan (Vellai *et al*., 2003) and rapamycin enhanced stress resistance and longevity in *C. elegans* by regulating SKN‐1/Nrf pathway (Robida‐Stubbs *et al*., 2012). Rapamycin regulated cell cycle arrest in mouse fibroblasts via the upregulation of Nrf2, but inhibited the phenotype of senescent cells in a Nrf2‐independent mechanism (Wang *et al*., 2017), indicating that although Nrf2 possesses the ability to combat the senescence cell, cell senescence is a complicated process and further studies on new anti‐aging compounds are still needed.

Oxidative stress and failure of the antioxidants’ protection are considered as 2 key contributors to age‐related cataract formation. LECs have powerful defense mechanisms against oxidation, with Nrf2 as a vital one, which can eliminate ROS, maintaining the redox balance. The basic level of Nrf2 in the lens is very low (von Otter *et al*., 2010). Overproduction of ROS leads to the suppression of Nrf2‐dependent antioxidant protection in LECs (Elanchezhian *et al*., 2012b); 2 haplotype alleles of NFE2L2 were found to be associated with cataract, indicating that NFE2L2 gene variation may affect cataract progression, although not convinced enough to support NFE2L2 or KEAP1 as cataract susceptibility genes (von Otter *et al*., 2010). A dramatically decreased level of Nrf2 (protein and gene), a significantly increased level of Keap1 (protein and gene), and an highly elevated levels of DNA demethylation in the Keap1 promoter were found in cultured HLECs, human aging lenses, and diabetic cataractous lenses (Palsamy *et al*., 2012). On the contrary, DNA methylation was found in the clear human lens and cultured HLECs (SRA01/04), indicating that DNA promoter demethylation of *Keap1* gene is an age‐dependent behavior, and it is also a crucial mechanism for cataract formation (Gao *et al*., 2015). The demethylation of the *Keap*1 promoter activates the expression of Keap1 protein, accelerates Nrf2 proteasomal degradation (Palsamy *et al*., 2012), and abolishes Nrf2 activity, leading to the failure of Nrf2‐dependent antioxidant system and resulting in the cataract formation eventually (Gao *et al*., 2015). These results further indicate that Nrf2 inducers might also serve as an anticataract formation compounds.

**Figure 1:**
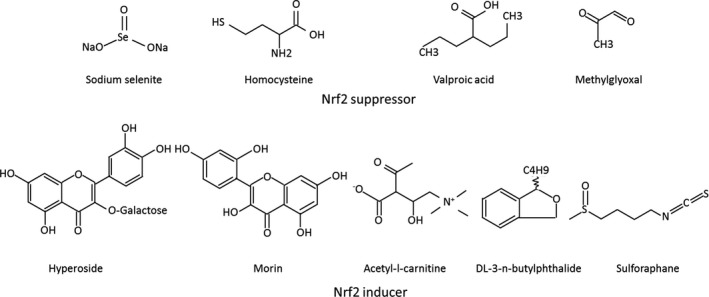
Chemical structure of typical Nrf2 suppressors and inducers tested in cataracts.

For better understanding the mechanism of cataract formation and well supporting the exploration of the anti‐aging medications in treating cataracts, here, we review recent scientific developments illustrating the relationship between Nrf2 and cataracts and discuss the future insight in this field. Sodium selenite, homocysteine (Hcy), valproic acid (VPA), lack/excessive O_2_, hypoglycemia under hypoxia, methylglyoxal (MGO) are reported as Nrf2 suppressors (Fig. 1), which aggravate cataract formation by inhibiting the Nrf2‐dependent antioxidant protection in lenses (Table 1). Hyperoside, morin, Acetyl‐l‐carnitine (ALCAR), sulforaphane (SFN), DL‐3‐n‐butylphthalide (NBP), and *Rosa laevigata Michx*. (RLM) extracts are reported as Nrf2 inducers (Fig. 1), which activate Nrf2 and protect lenses from oxidation (Table 1). These results indicate that Nrf2 serves as a promising therapeutic target for preventing and delaying the cataract formation.

**Table 1:** Studies on the relationship between Nrf2 inducer/suppressor and cataract models

Chemical	Type	Level	Models	Results	References
Hcy (−)	*In vitro*	Cell	HLECs treated with Hcy	High Hcy‐induced ROS overproduction, ER stress, UPR activation, Ca^2+^ releases and Nrf2 degradation in LECs	Elanchezhian *et al*. (2012a)
VPA (−)	*In vitro*	Cell	HLECs treated with VPA	VPA‐induced ROS overproduction, UPR activation, Ca^2+^ releases and suppressed the Nrf2/Keap1‐dependent catalase and GR expressions, resulting in HLECs death	Palsamy *et al*. (2014c)
Lack/excessive O_2_ (−)	*In vitro*	Cell	HLECs cultured under different O_2_	ROS, ER stress, keap1, cell death were increased and Nrf2 was decreased in HLECs cultured in 0 or 20% O_2_ than in 1 or 4% O_2_	Zheng *et al*. (2015)
Sodium selenite (−)	*In vitro*	Cell	HLECs treated with sodium selenite	Sodium selenite‐induced ROS overproduction, ER stress, UPR activation, Ca^2+^ releases, Keap1 overexpression and Nrf2 degradation, resulting in HLECs death	Palsamy *et al*. (2014b)
		Animal	Lenses enucleated from sodium selenite injected rats	Overproduction of ROS in LECs and newly formed lens fiber cells resulted in HLECs death	
Hypoglycemia under hypoxia (−)	*In vitro*	Cell	HLECs cultured under different O_2_	Hypoglycemia under hypoxia induced the UPR, promote cell proliferation and cause loss of GSH in HLECs	Elanchezhian *et al*. (2012b)
		Animal	Rat lenses organ cultured under different O_2_	UPR was activated by ischemia in rat lenses	
MGO (−)	*In vitro*	Cell	HLECs treated with MGO	MGO‐induced ROS overproduction, ER stress, UPR activation, Ca^2+^ releases, Keap1 overexpression and Nrf2 degradation, resulting in HLECs death	Palsamy *et al*. (2014a)
		Animal	Nrf2^−/−^, Nrf2^+/+^ diabetic mice lenses cultured with MGO	Greater ROS production and more cell death were found in Nrf2^−/−^ diabetic mouse LECs than those of Nrf2^+/+^ mouse	
	*In vivo*	Human	Human clear lenses and diabetic cataractous lenses	Clear lenses slowly lose 5‐methylcytosine in the Keap1 promoter at a rate of 1% per year. Diabetic cataractous lenses lost 90% of the 5 methylcytosine	
RLM extract (+)	*In vitro*	Cell	HLECs cultured under high glucose	RLM decreased ROS, elevated MMP protein in HLECs cultured under high glucose through the induction of HO‐1 expression via PI3K/AKT and Nrf2/ARE pathways	Liu *et al*. (2015)
Hyperoside (+)	*In vitro*	Cell	HLECs treated with hyperoside	Hyperoside increased the HO‐1 expression by activating ERK/Nrf2 pathway	Park *et al*. (2016)
Morin (+)	*In vitro*	Cell	HLECs treated with Morin	Morin increased the HO‐1 expression by activating ERK/Nrf2 pathway	Park *et al*. (2013)
ALCAR (+)	*In vitro*	Cell	HLECs treated with Hcy	ALCAR prevented Hcy‐induced‐ER stress, ROS overproduction, UPR activation and cell death in HLECs by activating Nrf2/Keap1 controlled catalase, superoxide dismutase, GPx, GSH expression	Yang *et al*. (2015)
SFN (+)	*In vitro*	Cell	HLECs treated with H_2_O_2_	SFN inhibited H_2_O_2_‐induced apoptosis in FHL124 cells by inducing Nrf2 nucleus translocation	Liu *et al*. (2013)
		Animal	Organ cultured porcine lenses	SFN protected against H_2_O_2_‐induced opacification	
		Animal	Organ cultured mouse lenses	SFN enhanced TrxR activity in mouse lens	Varma *et al*. (2013)
NBP (+)	*In vivo*	Animal	STZ‐induced diabetic cataract rat	NBP improved the cataract scores, increased 2, 4‐dinitrophenylhydrazone, 4‐hydroxynonenal, malondialdehyde, Nrf2, thioredoxin and catalase expression in the lens, and decreased blood glucose, serum malondialdehyde and 8‐Hydroxydeovexyguanosine	Wang *et al*. (2016)

Hcy, homocysteine; VPA, valproic acid; MGO, methylglyoxal; RLM, Rosa laevigata Michx; ALCAR, acetyl‐l‐carnitine; SFN, sulforaphane; NBP, DL‐3‐n‐butylphthalide. (−) indicates Nrf2 suppressor and (+) indicates Nrf2 inducer.

The PubMed database was searched on January 19, 2017 with the terms ‘cataract*’, ‘lens*’, ‘nuclear factor erythroid 2‐related factor 2’, ‘Nrf2’, either alone and in combination. All relevant articles were manually picked out, with the most related 24 papers covering the period of 2002–2017.

## Nrf2 suppressors and cataracts

### Sodium selenite

Selenium is an essential micronutrient that involves in various important biological activities. Superfluous selenium, exposure at high levels (>1 μm), was considered to pose a great toxic threat to organisms for its pro‐oxidant effect. As elucidated in our previous review (Liu *et al*., 2017a), selenium‐ or sodium selenite‐induced cataract in neonatal rats is the well‐known cataract animal models. In lenses enucleated from sodium selenite‐treated rats, ROS overproduction caused considerable LECs death and cataract formation (Palsamy *et al*., 2014b). In HLECs, exposure to sodium selenite elevated ER stress activated unfolded protein response (UPR), increased endoplasmic reticulum (ER)‐Ca^2+^‐release, generated ROS, and demethylated Keap1 DNA promoter, leading to the cell death. Keap1 DNA demethylation of Keap1 promoter increased the Keap1 protein transcription. Overexpression Keap1 protein inhibited the Nrf2 protein via ER‐associated degradation, resulting in the inhibition of Nrf2/Keap1‐dependent antioxidant systems in the sodium selenite‐treated HLECs. Eventually, cataract formed, as the cellular redox status in lens, is altered toward oxidation (Palsamy *et al*., 2014b). However, further studies should be designed to determine the basis of the putative effect of ER stressors merely on immature LECs as well as explore the mechanism by which the DNA in these cells degenerate following selenite exposure.

### VPA

Known as an anticonvulsant drug, VPA, *in utero* exposure, was also found to induce congenital cataracts (Glover *et al*., 2002). Promoter DNA demethylation of *Keap1* was involved in human cataract formation by suppressing Nrf2‐dependent antioxidant system (Gao *et al*., 2015). VPA could stimulate ROS production, leading to the transcription of ARE‐driven genes (Kawai & Arinze, 2006). VPA induced ER stress and unfolded protein response (UPR) by activation the ER stress sensor proteins and transcription factor 6 in HLECs, resulting in DNA demethylation of the Keap1 promoter (Palsamy *et al*., 2014c), which suppresses the Nrf2‐dependent antioxidant system and results in cataract formation.

### Lack/excessive O_2_

The lens is located in a hypoxic environment (0.5–2.3% O_2_) under normal circumstance (Shui *et al*., 2006). O_2_ level with the range of 0.1–1.0% can be defined as severe hypoxia, and the O_2_ level in lens can effortlessly fall into the severe hypoxia range even in a normal (13–15%) atmospheric O_2_ supply (Shui & Beebe, 2008), especially for diabetic patients (Holekamp *et al*., 2006). Either 0 or 20% atmospheric O_2_ induced significant ROS production and cell death in cultured HLECs, accompanied by increased UPR protein and inhibition of the Nrf2/Keap1‐dependent antioxidant protection in LECs, and the results showed that 1% O_2_ was appropriate for culturing LECs (Zheng *et al*., 2015).

### Hypoglycemia under hypoxia

Besides hypoxia, the lens was also located in a hypoglycemic environment with the glucose detected in the aqueous fluid of 2–4 mm and 1.6 mm in the vitreous fluid of human eyes (Lundquist & Osterlin, 1994). The lens can develop nuclear cataract rapidly, if glucose is deprived for 48 h (Chylack & Schaefer, 1976). In opposite, extracellular glucose accumulation may also trigger the hyperglycemic toxic effects, and aging lens with diabetes might aggravate these stresses with increased incidence for cataract formation and progression (Vinson, 2006). Considering the fact that lens is an insulin‐independent tissue and not capable to downregulate the glucose transport, the strict application of insulin unavoidably increases the incidence of severe hypoglycemia fourfold to sixfold (Lacherade *et al*., 2009). Low level of glucose under severe hypoxia induced the production of ROS, UPR activation and inhibited Nrf2/Keap1‐dependent antioxidant enzymes, resulting the LECs apoptosis, revealing a new connection between hypoglycemia under hypoxia and LECs function impairment (Elanchezhian *et al*., 2012b).

### MGO

DNA methylation was found to be completely methylated in HLEC line and clear human lens (Palsamy *et al*., 2012), while the demethylation of *Keap1* promoter DNA was found to be increased in diabetic ARCs (Palsamy *et al*., 2014a), indicating DNA demethylation of *keap1* may be involved in cataract formation. Streptozocin (STZ)‐induced diabetic Nrf2^−/−^ mice exhibited more cell death in lenses and developed cataract earlier than Nrf2^−/−^ mice (Palsamy *et al*., 2014a). As one of the abnormal reactive aldehydes that generated by hyperglycemia (Palsamy *et al*., 2014a), MGO induced oxidative stress in cultured HLECs and inhibited Nrf2/Keap1‐regulated GR, catalase protein expression, which eventually changed cellular redox balance toward lens oxidation and resulted in cataract formation (Palsamy *et al*., 2014a).

### Hcy

Hcy is biosynthesized from methionine in a multiple step pathway. An elevated level of serum Hcy is related to the formation of juvenile cataracts and ARCs (Sen *et al*., 2008). Recently, several studies have illustrated significant increase of Hcy in the plasma of ARCs’ patients (Sen *et al*., 2008). Elevated level of Hcy also increased protein misfolding in the ER, which led to the UPR (Ron & Walter, 2007; Todd *et al*., 2008). In agreement with these studies, it was also confirmed that Hcy (100 μm) could induce the generation of ROS and cell death in HLECs (Yang *et al*., 2015). This study revealed Hcy had the ability to induce the activation of UPR regulated by ER stress, overproduction of ROS, and inhibition of Nrf2/Keap1‐mediated antioxidant protection via demethylation of Keap1 promoter, resulting in cell death in HLECs (Elanchezhian *et al*., 2012a; Yang *et al*., 2015).

## Nrf2 inducers and cataracts

### ALCAR

Beside the well‐known antioxidant and anti‐inflammation effects, ALCAR (the acetyl ester of the trimethylated amino acid L‐carnitine) has also been reported to possess the ability to prevent apoptosis and aging in yeast (Palermo *et al*., 2010). Moreover, ALCAR has been discovered to prevent the cataract formation in rat models (Elanchezhian *et al*., 2007). Elanchezhian *et al*. (2007) also found the protective function of ALCAR in selenite‐induced cataracts in rat. ALCAR increased Nrf2‐regulated antioxidant proteins and decreased ER stress‐mediated proteins in Hcy‐treated cells. A similar effect was also found in expression of the *Nrf2* and *Keap1* gene (Yang *et al*., 2015). These results implied that ALCAR is a promising agent in defending the human lens against oxidative stress‐induced injury, thus preventing the cataract formation (Yang *et al*., 2015). However, further advanced studies are still needed for the development of ALCAR as an anticataract component.

### Hyperoside

Hyperoside (quercetin‐3‐D‐galactoside) generally found in *Hypericum perforatum* L., is a kind of flavonoid, which can inhibit the oxidative stress. Heme oxygenase‐1 (HO‐1), also known as 32‐kDa heat‐shock protein, is one of important enzymatic antioxidants involved in defense systems. Several signaling molecules are regulating the initiation of HO‐1, and Nrf2 is a key transcription factor of HO‐1. Hyperoside increased the expression of HO‐1, Nrf2, and its antioxidant response by upregulating extracellular signal‐regulated kinase (ERK) activity in hydrogen peroxide (H_2_O_2_)‐treated HLECs. Although not any anti‐aging function reported yet, hyperoside is an effective flavonoid to protect HLECs against oxidative stress through the induction of HO‐1 (Park *et al*., 2016).

### Morin

Morin is a type of flavonoids, also known as 3, 5, 7, 2′, 4′‐pentahydroxyflavone, and has been widely used as herbal medicines. Morin has many biological functions, including antioxidant (Zhang *et al*., 2009, 2011), anti‐inflammatory (Lee *et al*., 2016), antidiabetic osteopenia (Abuohashish *et al*., 2013), and neuroprotective effects (Lee *et al*., 2016). It has been reported that morin could protect cells against oxidative stress stimulated by H_2_O_2_ and γ‐ray radiation (Zhang *et al*., 2009, 2011). Recently, Park *et al*. confirmed that morin could stimulate ERK‐Nrf2 signaling pathway in HLECs (HLE‐B3), resulting in the upregulation of HO‐1 and cytoprotective effects against oxidative stress. Morin increased the protein level of Nrf2, which enhances the expression of HO‐1 via binding to the ARE. Besides, morin activated ERK and triggered transport from cytosol to nucleus (Park *et al*., 2013).

### SFN

SFN, a plant‐extracted isothiocyanate 1‐isothiocyanato‐4‐methylsulfinylbutane, induced the transcription of some antioxidant enzymes and stimulates cellular antioxidant defenses. Nevertheless, the mechanism involved is still not fully understood. TrxR is efficient in inhibiting oxidative stress with accumulating evidence of its value in preventing cataract formation, when consumed nutritionally. Recently, SFN has been detected to increase the activity of TrxR in the mouse lens, and prevents the tissue against oxidative stress that contributes to cataract formation. Further researches on the behaviors of other antioxidant enzymes like quinone oxidoreductase are still in progress (Varma *et al*., 2013). SFN was found to significantly inhibit the increased cytotoxicity/apoptosis induced by H_2_O_2_ in the human lens epithelial cell line FHL124 (Liu *et al*., 2013). Experimental evidence suggested that SFN could shield against H_2_O_2_‐stimulated opacification in cultured porcine lenses. Nrf2 translocated to the nucleus followed by the exposure of 0.5–2.0 μm SFN. Dietary SFN presents the capability to guard human lens cells against ROS via induction of the Keap1‐Nrf2‐ARE pathway and might postpone the formation of cataract (Liu *et al*., 2013). Furthermore, SFN supplement also reduced aging‐related oxidative stress and neuro‐inflammation in microglia cells both *in vitro* and *in vivo* (Townsend & Johnson, 2016), as well as protected the UV‐induced extrinsic skin aging (Sikdar *et al*., 2016). These results indicate that SFN may serve as a very promising anti‐aging compound in future.

### NBP

NBP, a multiple‐target neuroprotective drug, is widely used clinically for the treatment of ischemic stroke, which greatly decreases oxidative damages, increases mitochondrial function, reduces inflammation, and attenuates neuronal apoptosis. NBP has been indicated to upregulate transcriptional activity and subsequently exert an antioxidant activity in the model of amyotrophic lateral sclerosis (Feng *et al*., 2012). In a diabetic rat model, NBP was found to increase the expression of Nrf2 in the lens of diabetic rats and decrease levels of blood glucose, serum MDA, and 8‐OHdG. NBP‐treated mice were also shown improvement in oxidative stress, as indicated by the levels of DNP, 4‐HNE, and MDA in lens. Even though the precise mechanisms of NBP in preventing diabetic cataract are still needed to investigate, NBP might be a promising therapeutic drug to prevent or treat diabetic cataract (Wang *et al*., 2016).

### RLM

Diabetic cataract is one of the most serious complications of diabetes. Accumulating evidence reveals that ROS overproduced in mitochondria can induce metabolic abnormality, which might be a significant factor in the formation of diabetic cataract. RLM, belonging to the *Rosaceae* family, has been used as medicinal plants in China for centuries. RLM extract possess antioxidant and free radical‐scavenging capacity. The antioxidant effect of RLM on diabetic cataract was investigated in immortalized LEC lines (SRA01/04) cultured with 5.5 mm high glucose (Liu *et al*., 2015). This study indicated that RLM could reduce the production of ROS and improve mitochondrial membrane potential (MMP), via the stimulation of HO‐1 expression, in SRA01/04 cells in the hyperglycemic state. Moreover, the protective effects of RLM are regulated through the PI3K/serine–threonine kinase (AKT) and Nrf2/ARE signaling pathways (Liu *et al*., 2015). Nevertheless, additional studies should explore the mechanism of HO‐1 against the oxidative stress in diabetic cataract.

## Conclusions

**Figure 2:**
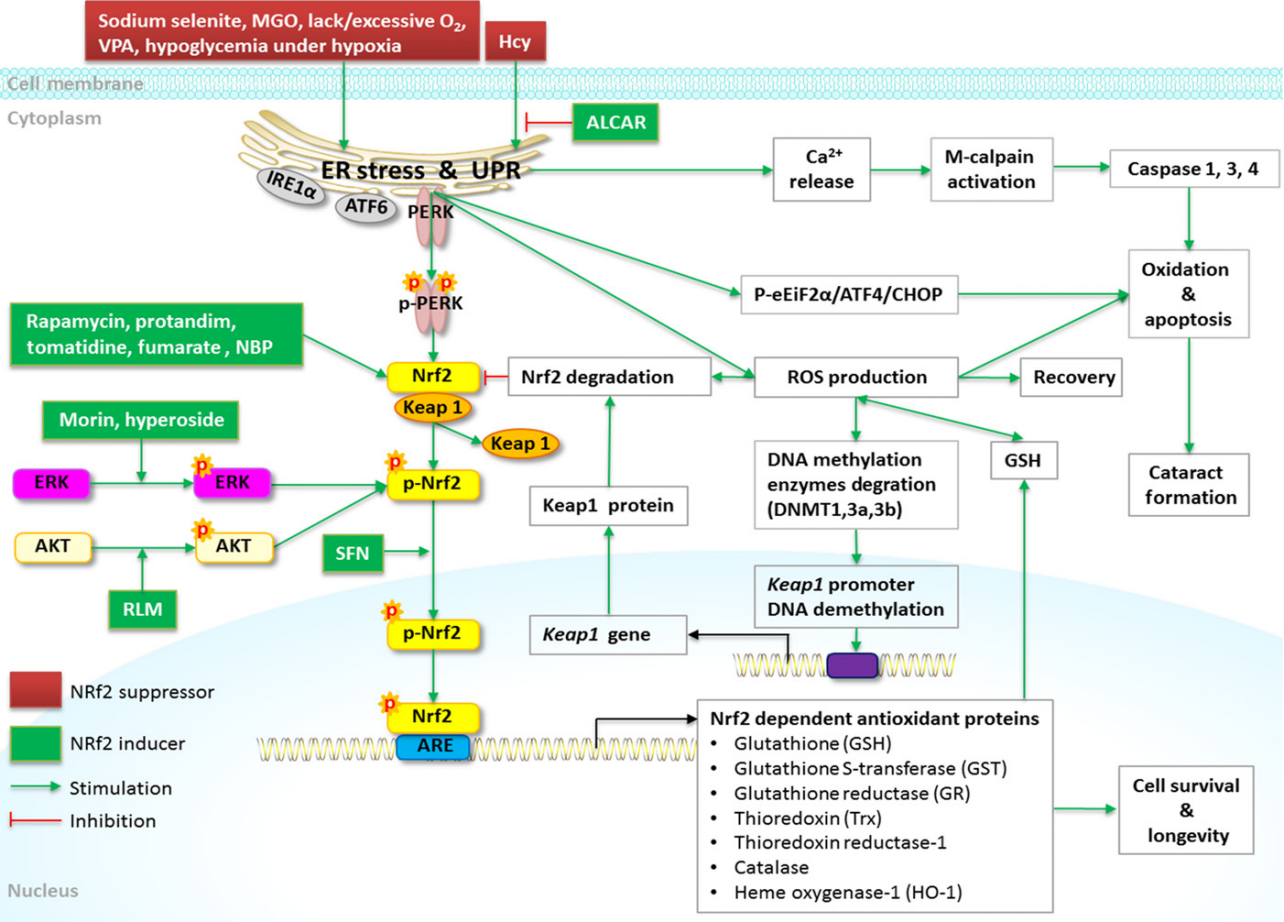
Nrf2 signaling and regulation in the lens. Various cataractogenic stressors induce ER stress, UPR activation, P‐PERK expression, and Nrf2 phosphorylation. The phosphorylated Nrf2 separates from Keap1, binds with ARE in the nucleus, and initiates the antioxidant enzymes transcriptions (GSH, GST, GR, Trx, thioredoxin reductase‐1, catalase, HO‐1), which help eliminating ROS by regenerating GSH. Severe or prolonged ER stressors (sodium selenite, Hcy, VPA, lack/excessive O_2_, hypoglycemia under hypoxia) cause chronic apoptotic UPR and ROS overproduction, ER‐Ca^2+^ release, calpain overexpression, and caspase 1,3,4 pathways’ activation, leading to the lens oxidation and cell death. Chronic apoptotic UPR also suppresses the Nrf2‐dependent antioxidant protection resulting in cataract formation. Hyperoside, morin, acetyl‐l‐carnitine, DL‐3‐n‐butylphthalide, RLM activate Nrf2 and protect lenses from oxidation. Excessive ROS also inhibit the Nrf2‐dependent antioxidant system via accelerating the DNA methylation enzymes degradation, triggering demethylation of DNA in the *Keap1* promoter and overexpression of Keap1, which enhances the Nrf2 proteasomal degradation. Rapamycin, protandim, tomatidine, fumarate activate the Nrf2 signaling and extend the life longevity. Green solid line indicates direct stimulation. Red solid line indicates direct inhibition. ROS, reactive oxygen species; Nrf2, transcription factors like nuclear factor (erythroid‐ derived 2)‐like 2; Keap1, Kelch‐like erythroid‐cell‐derived protein with CNC homology (ECH)‐associated protein 1; ARE, antioxidant response element; GST, glutathione‐S‐transferase; GR, glutathione reductase; p‐PERK, phosphorylated protein kinase RNA (PKR)‐like endoplasmic reticulum kinase; UPR, unfolded protein response; ER, endoplasmic reticulum; Hcy, homocysteine; Trx, thioredoxin; GSH, glutathione; VPA, valproic acid; ALCAR, acetyl‐l‐carnitine; p‐eIF2α, phosphorylated eukaryotic initiation factor 2α; IRE1α, Inositol‐requiring kinase 1α; ATF6, transcription factor 6; ATF4, activating transcription factor 4; CHOP, CCAAT/enhancer‐binding protein‐homologous protein; Dnmt3a, DNA methyltransferase 3a; Dnmt3b, DNA methyltransferase 3b; Dnmt1, DNA methyltransferase 1; HO‐1, heme oxygenase‐1; ERK, extracellular signal‐regulated kinase; SFN, sulforaphane; NBP, DL‐3‐n‐butylphthalide; MGO, methylglyoxal; RLM, Rosa laevigata Michx.; AKT, serine–threonine kinase.

Accumulating evidence indicates that oxidative stress increases the injuries in the lens cell through oxidation of proteins, DNA damage, membrane lipid peroxidation, ER stress, UPR activation, and imbalance in calcium homeostasis, etc., all of which contribute to cataractogensis. The Nrf2/Keap1/ARE signaling system is now regarded as one of the major cell defense mechanisms against oxidative stresses. Hence, the stimulation of the Nrf2‐ARE signaling pathway has been evaluated as a crucial target for the design and synthesis of new agents for cataracts. Recently, natural compounds are a key source of Nrf2 inducers, which have been extensively studied. Agents in this article reviewed have illustrated most remarkable effects *in vitro* and *in vivo* models, protecting HLECs against oxidative stress, reducing the aberrant proteins and preventing the formation of cataract (Fig. 2). All those promising effects have been associated with the antioxidant and Nrf2 inducing effects of the compounds studied. In conclusion, the Nrf2/Keap1/ARE signaling pathway is a promising preventive and therapeutic target against oxidative stress for cataracts. Nrf2 inducers should be considered as an excellent target to start clinical trials.

## Author contributions

The topic was conceptualized by CWL. XFL, and DDZ, and JLH contributed to the literature database search, and writing of the manuscript. TX, CBL, CL, and CS contributed to vital revising. THM contributes to English Polishing.

## Funding

It was funded by Development and Reform Commission of Jilin Province [2015Y031‐1] and The First Hospital of Jilin University Grant [JDYY72016055].

## Conflicts of interest

None declared.
